# Supplementary material for: Gestational choline supplementation regulates hippocampal granule neuron development and emotion-like behavior
Source: Commun Biol. 2026 Apr 2;9:731. doi: 10.1038/s42003-026-09955-7 (PMC13219442; doi:10.1038/s42003-026-09955-7)
Supplement: Supplementary file 2 — Supplementary Information [file 42003_2026_9955_MOESM2_ESM.pdf]

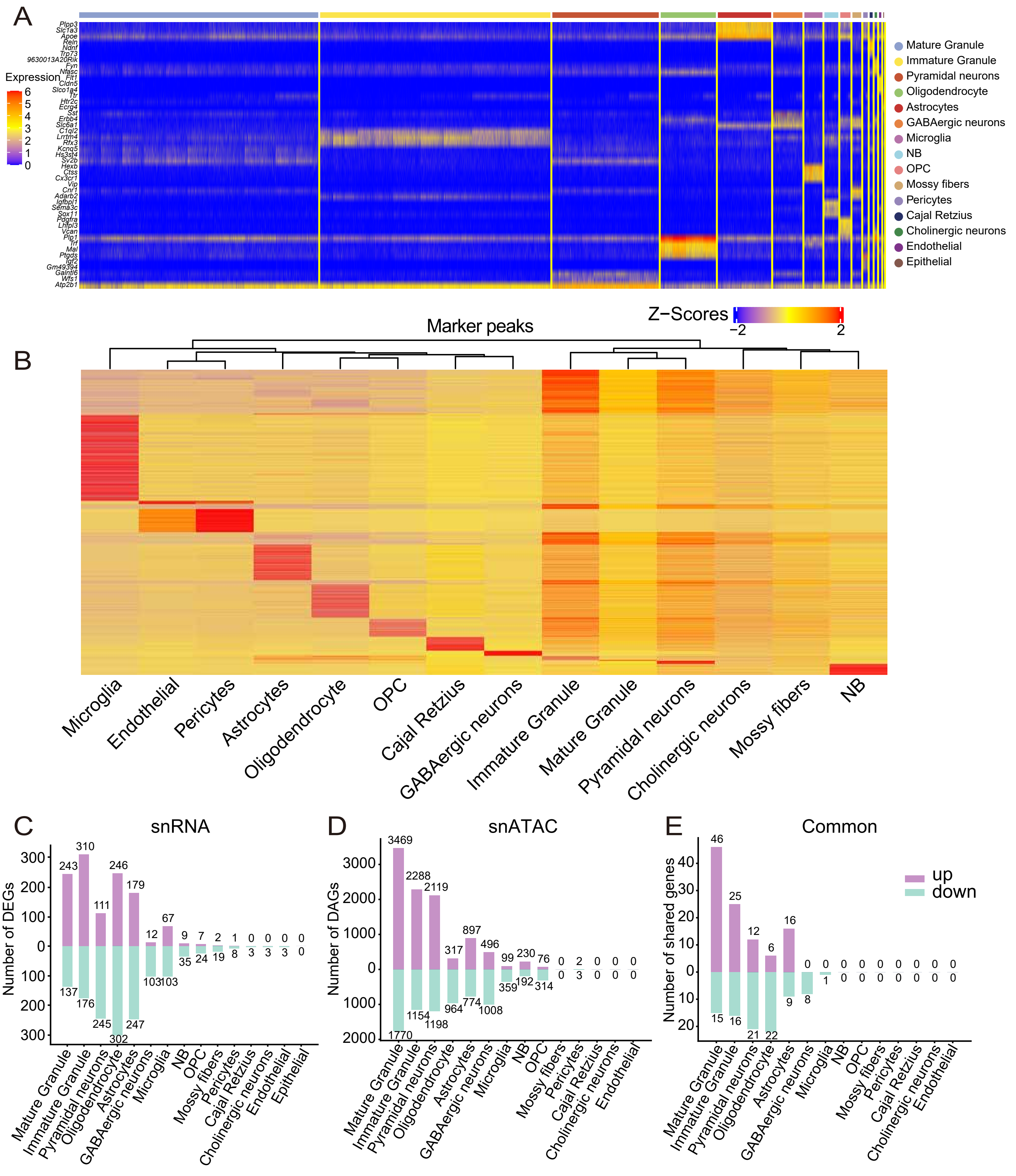

Fig. S1: Multimodal atlas of the hippocampi of $F1_{CON}$ and $F1_{GCS}$ male mice at P60. **A** Heatmap showing the expressions of the top three marker genes per cell type in snRNA-Seq data. **B** Heatmap of marker peaks (differentially accessible peaks) from snATAC-Seq for the indicated cell types. The color scale represents the relative Z-score of each marker peak. **C–E** Number of up-regulated (upper) and down-regulated (lower) differentially expressed genes (DEGs, C) and differentially accessible genes (DAGs, D) across distinct cell types, identified by snRNA-Seq and snATAC-Seq, and those shared between the two sequencing approaches (E).

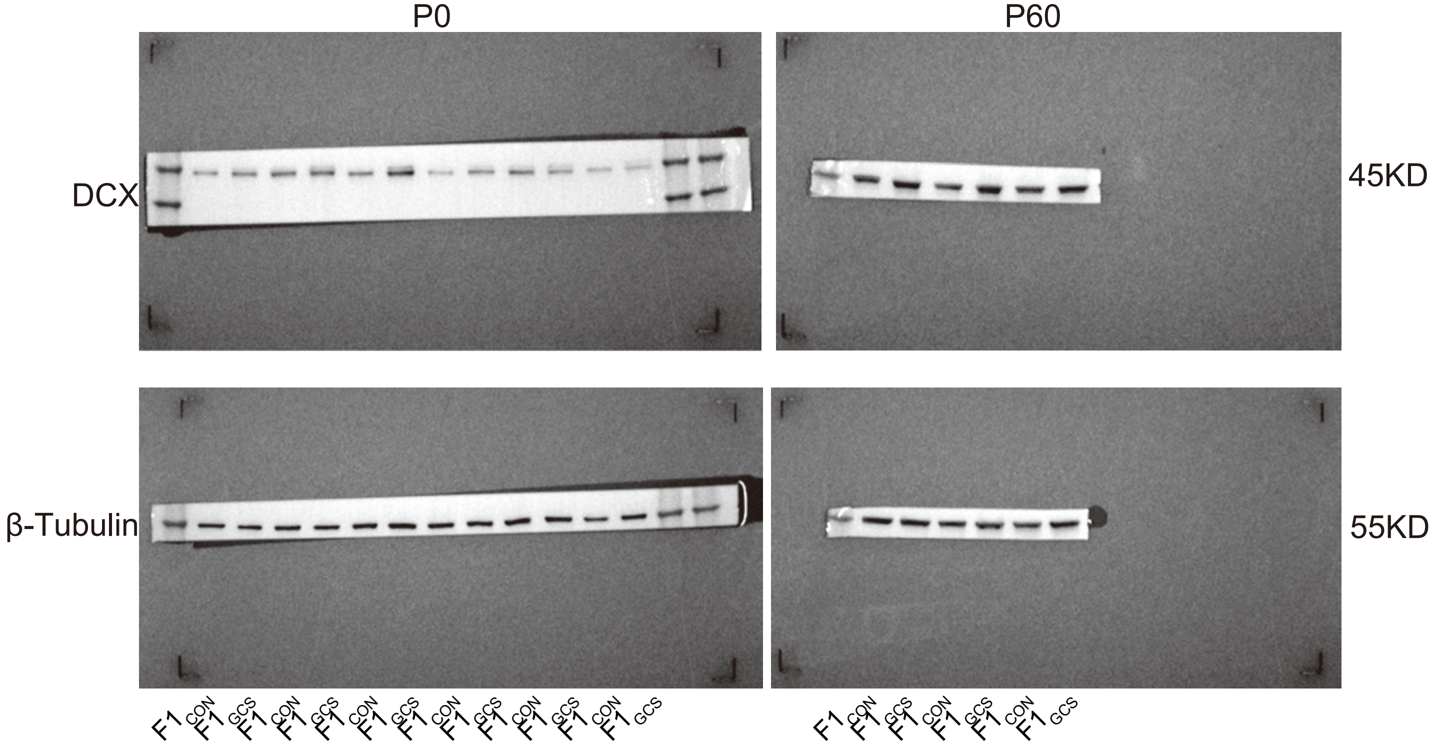

Fig. S2: Uncropped and unedited images of immunoblots showing DCX protein levels in the hippocampus of $F1_{CON}$ and $F1_{GCS}$ mice at P0 and P60.

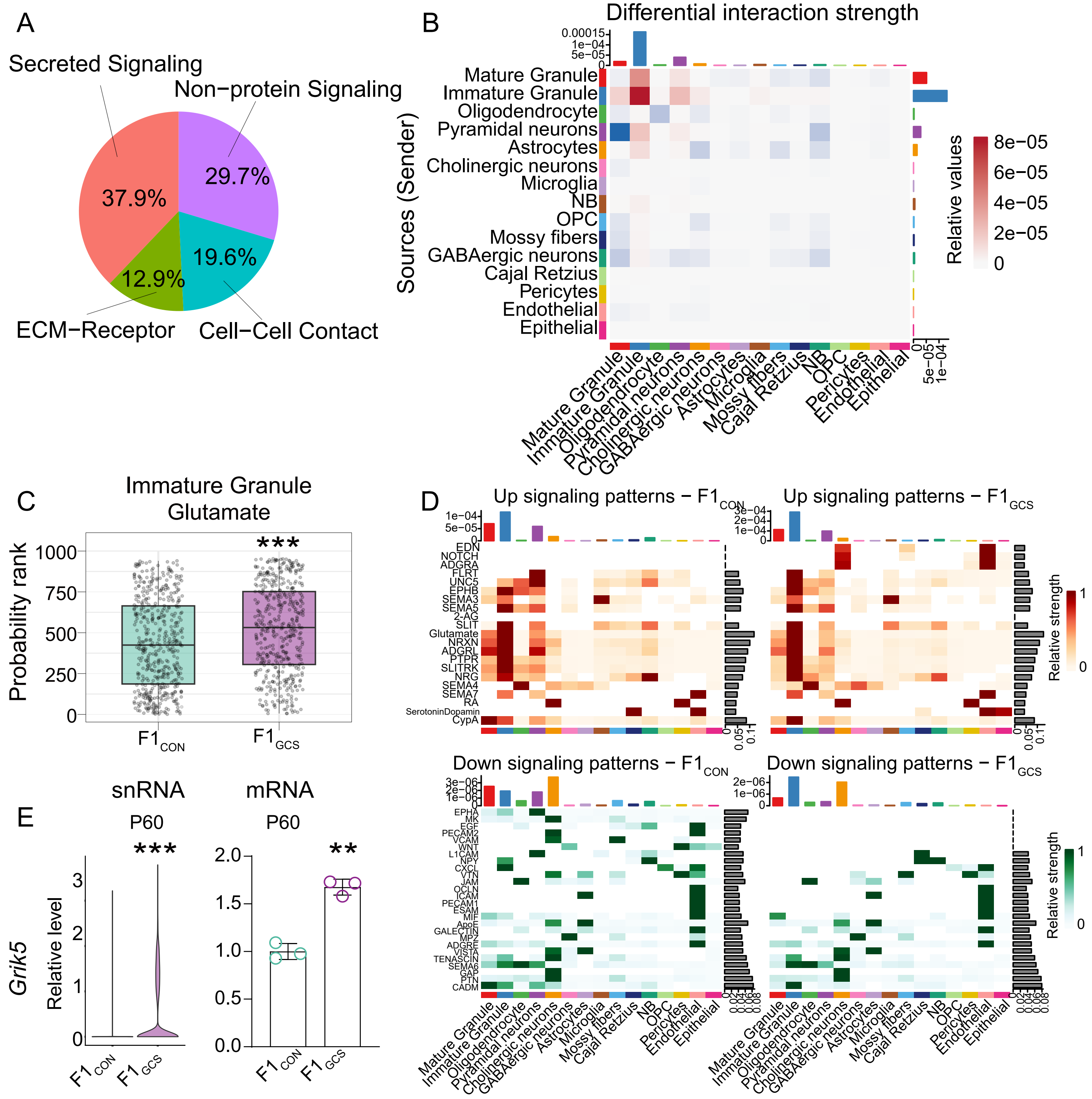

Fig. S3: Cell communication analysis of $F1_{CON}$ and $F1_{GCS}$ mice hippocampus at P60. **A** Proportion of ligand-receptor (LR) pairs corresponding to four intercellular communication patterns in the CellChat database. **B** Heatmap showing changes in intercellular and intracellular communication strength among 15 hippocampal cell types between $F1_{CON}$ and $F1_{GCS}$ mice at P60. In the color bar, red indicates enhanced signaling in $F1_{GCS}$ relative to $F1_{CON}$, while blue indicates weakened signaling. **C** Cell communication probability rank of the glutamate pathway in immature granule neurons of $F1_{CON}$ (green) and $F1_{GCS}$ (purple) mice. Colors represent groups, and asterisks with different quantities indicate significance levels (* $p < 0.05$; ** $p < 0.01$; *** $p < 0.001$). For box plots, the boxes represent the upper and lower quartiles, and the horizontal lines within represent the medians. **D** Relative communication strengths among 15 cell types in $F1_{CON}$ and $F1_{GCS}$ mice, specifically for signaling pathways that are enhanced (upper, red) or weakened (lower, green) in $F1_{GCS}$. **E** Left: Violin plots showing the relative expression of *Grik5* (adjusted p = 3.829e-31, $\log_2$[fold change] (FC) ($\log_2$FC) = 0.344) in immature granule neurons from $F1_{CON}$ and $F1_{GCS}$ mice. Right: Validation of the levels of *Grik5* (p = 0.005) at P60 using the qRT-PCR approach. Mann–Whitney U test. ** (adjusted) $p < 0.01$; *** (adjusted) $p < 0.001$. Data are represented as means ± SEMs.

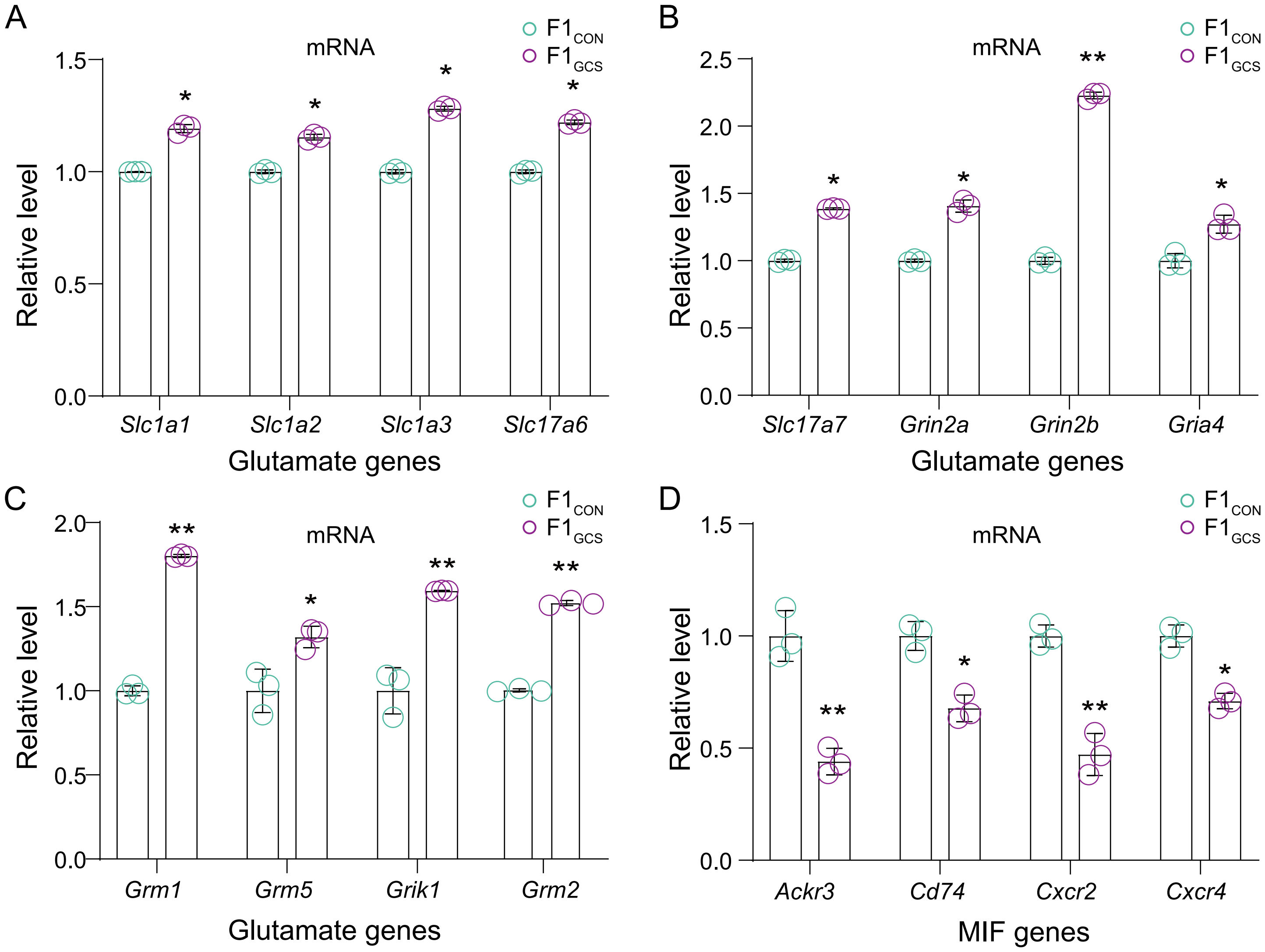

Fig. S4: Expression levels of glutamate- and MIF-related genes in the hippocampus of $F1_{CON}$ and $F1_{GCS}$ mice at P0 validated by qRT-PCR. **A–C** The expression levels of glutamate-related genes were significantly up-regulated in the hippocampus of $F1_{GCS}$ mice at P0. **D** The expression levels of MIF-related genes were significantly down-regulated in the hippocampus of $F1_{GCS}$ mice at P0. Mann–Whitney U test was performed for all the above genes. * $p < 0.05$; ** $p < 0.01$. Data are represented as means ± SEMs.

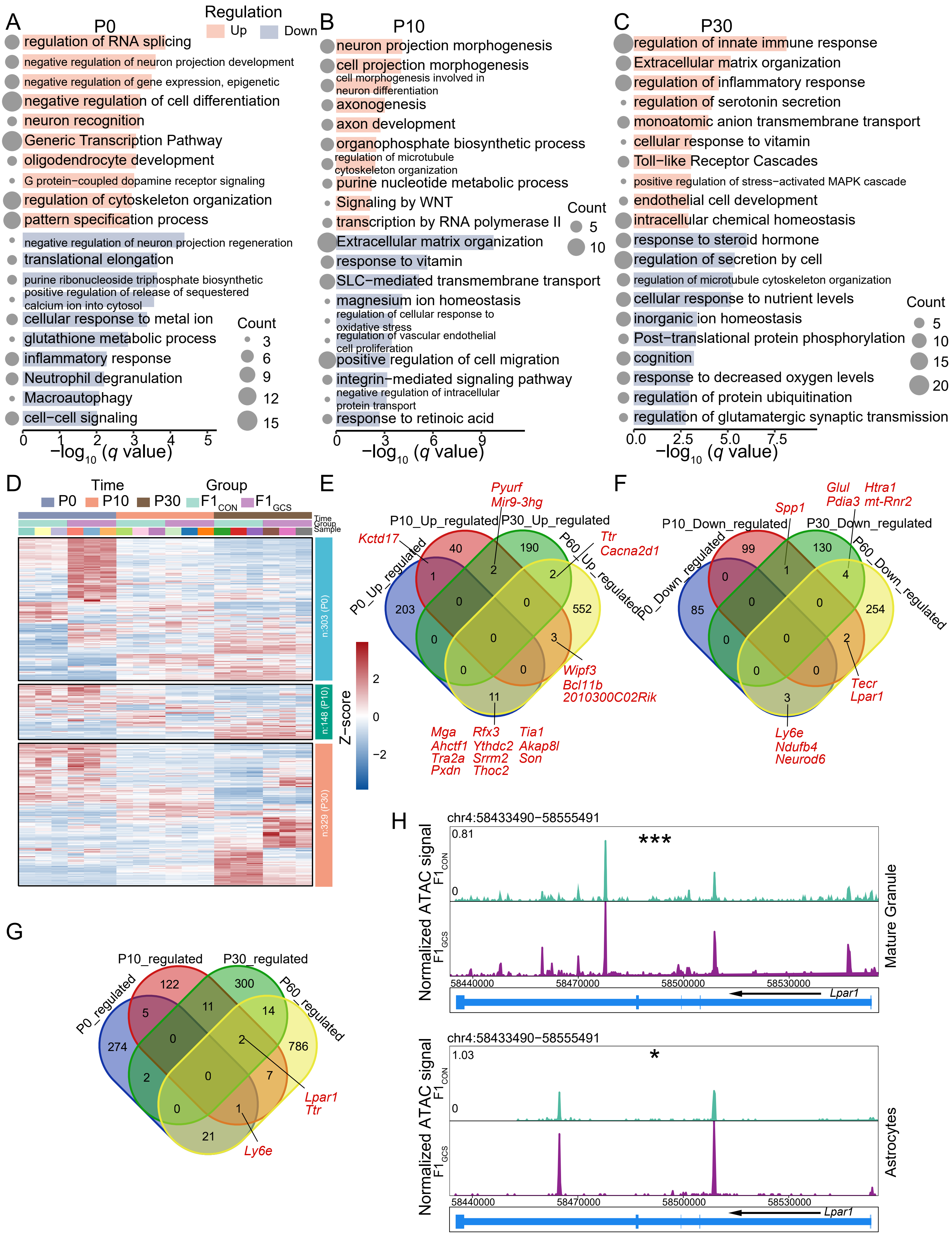

A
P0
Regulation
Up
Down
regulation of RNA splicing
negative regulation of neuron projection development
negative regulation of gene expression, epigenetic
negative regulation of cell differentiation
neuron recognition
Generic Transcription Pathway
oligodendrocyte development
G protein−coupled dopamine receptor signaling
regulation of cytoskeleton organization
pattern specification process
negative regulation of neuron projection regeneration
translational elongation
purine ribonucleoside triphosphate biosynthetic
positive regulation of release of sequestered calcium ion into cytosol
cellular response to metal ion
glutathione metabolic process
inflammatory response
Neutrophil degranulation
Macroautophagy
cell−cell signaling
Count
3
6
9
12
15
0 1 2 3 4 5
−log10 (q value)
B
P10
neuron projection morphogenesis
cell projection morphogenesis
cell morphogenesis involved in neuron differentiation
axonogenesis
axon development
organophosphate biosynthetic process
regulation of microtubule cytoskeleton organization
purine nucleotide metabolic process
Signaling by WNT
transcription by RNA polymerase II
Extracellular matrix organization
response to vitamin
SLC−mediated transmembrane transport
magnesium ion homeostasis
regulation of cellular response to oxidative stress
regulation of vascular endothelial cell proliferation
positive regulation of cell migration
integrin−mediated signaling pathway
negative regulation of intracellular protein transport
response to retinoic acid
Count
5
10
0 3 6 9
−log10 (q value)
C
P30
regulation of innate immune response
Extracellular matrix organization
regulation of inflammatory response
regulation of serotonin secretion
monoatomic anion transmembrane transport
cellular response to vitamin
Toll−like Receptor Cascades
positive regulation of stress−activated MAPK cascade
endothelial cell development
intracellular chemical homeostasis
response to steroid hormone
regulation of secretion by cell
regulation of microtubule cytoskeleton organization
cellular response to nutrient levels
inorganic ion homeostasis
Post−translational protein phosphorylation
cognition
response to decreased oxygen levels
regulation of protein ubiquitination
regulation of glutamatergic synaptic transmission
Count
5
10
15
20
0.0 2.5 5.0 7.5
−log10 (q value)
D
Time
P0 P10 P30
Group
F1CON F1GCS
Time
Group
Sample
n:303 (P0)
n:148 (P10)
n:329 (P30)
Z−score
2
0
−2
E
P10_Up_regulated
P30_Up_regulated
P0_Up_regulated
P60_Up_regulated
Pyurf
Mir9-3hg
Kctd17
Ttr
Cacna2d1
40 190 1 2 2 203 0 0 552 0 0 3 0 0 11
Wipf3
Bcl11b
2010300C02Rik
Mga Ahctf1 Tra2a Pxdn
Rfx3 Ythdc2 Srrm2 Thoc2
Tia1 Akap8l Son
F
P10_Down_regulated
P30_Down_regulated
P0_Down_regulated
P60_Down_regulated
Glul Pdia3
Htra1 mt-Rnr2
Spp1
99 130 0 1 4 85 0 0 254 0 0 2 0 0 3
Tecr
Lpar1
Ly6e
Ndufb4
Neurod6
G
P10_regulated
P30_regulated
P0_regulated
P60_regulated
122 300 5 11 14 274 0 2 786 2 0 7 0 1 21
Lpar1
Ttr
Ly6e
H
chr4:58433490−58555491
0.81
***
0
F1CON
F1GCS
Normalized ATAC signal
Mature Granule
58440000 58470000 58500000 58530000
Lpar1
chr4:58433490−58555491
1.03
*
0
F1CON
F1GCS
Normalized ATAC signal
Astrocytes
58440000 58470000 58500000 58530000
Lpar1

Fig. S5: RNA-Seq and snRNA-Seq analysis of differentially expressed gene enrichment pathways, expression distributions, and gene set overlaps in the hippocampus of $F1_{CON}$ and $F1_{GCS}$ mice at P0, P10, P30, and P60. **A–C** Pathways enriched by differentially expressed genes in the hippocampus of $F1_{GCS}$ at P0 (A), P10 (B), and P30 (C), identified via RNA-Seq analysis. Circle size corresponds to the number of genes enriched in each pathway. **D** Expression distributions of DEGs in the hippocampus of $F1_{CON}$ and $F1_{GCS}$ mice at P0, P10, and P30. The heatmap presents relative gene expression levels (Z-score). **E–G** Overlap of up-regulated (E), down-regulated (F), and all (G) differentially expressed genes in the hippocampus of $F1_{CON}$ and $F1_{GCS}$ mice at P0, P10, P30, and P60. **H** Genome browser tracks showing single-nucleus chromatin accessibility at the *Lpar1* locus in mature granule neurons (FDR = 3.08e-09, log_2FC = 0.490) and astrocytes (FDR = 0.048, log_2FC = 0.818) between $F1_{CON}$ and $F1_{GCS}$.

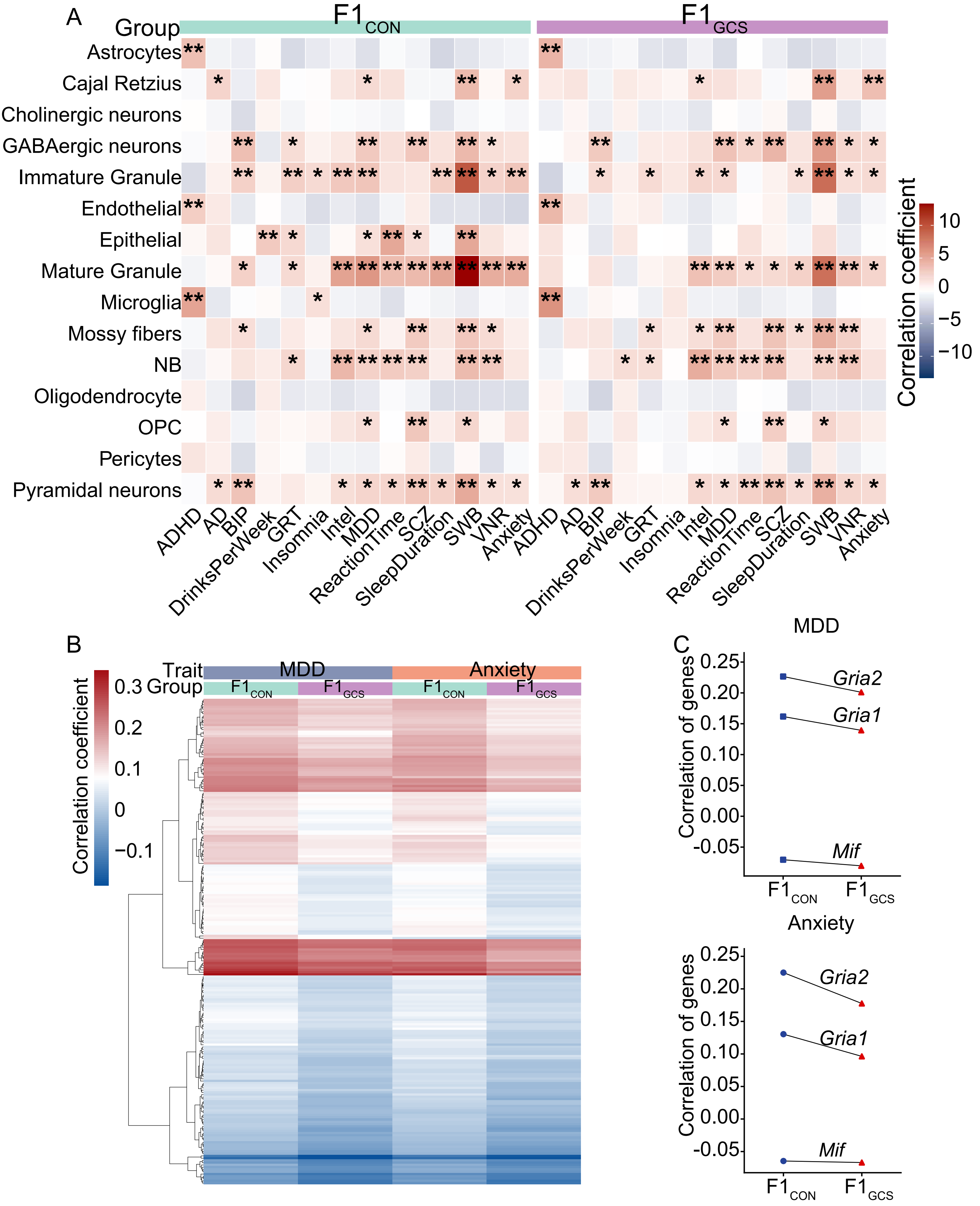
A
Group
F1CON
F1GCS
Astrocytes
Cajal Retzius
Cholinergic neurons
GABAergic neurons
Immature Granule
Endothelial
Epithelial
Mature Granule
Microglia
Mossy fibers
NB
Oligodendrocyte
OPC
Pericytes
Pyramidal neurons
ADHD
AD
BIP
DrinksPerWeek
GRT
Insomnia
Intel
MDD
ReactionTime
SCZ
SleepDuration
SWB
VNR
Anxiety
Correlation coefficient
10
5
0
−5
−10
B
Trait
Group
MDD
Anxiety
F1CON
F1GCS
Correlation coefficient
0.3
0.2
0.1
0
−0.1
C
MDD
Anxiety
Correlation of genes
0.25
0.20
0.15
0.10
0.05
0.00
-0.05
Gria2
Gria1
Mif
F1CON
F1GCS

Fig. S6: GCS reduces correlation of mouse hippocampus with MDD and anxiety. **A** scDRS correlation coefficients of 15 cell types in $F1_{CON}$ and $F1_{GCS}$ with respect to diseases and traits, including attention deficit hyperactivity disorder (ADHD), Alzheimer's disease (AD), bipolar disorder (BIP), drinks per week, general risk tolerance (GRT), insomnia, intelligence (Intel), major depressive disorder (MDD), reaction time, schizophrenia (SCZ), sleep duration, subject well-being (SWB), verbal numerical reasoning (VNR), and anxiety. The color bar indicates correlation coefficients, and asterisks denote significance. **B** Correlation coefficients in $F1_{CON}$ and $F1_{GCS}$ for the top 300 genes with the most significant reduction in correlation with MDD and anxiety in $F1_{GCS}$, without distinguishing cell populations. **C** Correlation coefficients of *Mif*, *Gria1*, and *Gria2* genes with MDD and anxiety across all hippocampal cell types in $F1_{CON}$ and $F1_{GCS}$.
