# Supplementary material for: Gestational choline supplementation regulates hippocampal granule neuron development and emotion-like behavior
Source: Commun Biol. 2026 Apr 2;9:731. doi: 10.1038/s42003-026-09955-7 (PMC13219442; doi:10.1038/s42003-026-09955-7)
Supplement: Supplementary file 3 — Description of Additional Supplementary Files [file 42003_2026_9955_MOESM3_ESM.pdf]

Description of Additional Supplementary Files

File name: Supplementary Data 1

Description: Cell distribution in the hippocampus of $F1_{CON}$ and $F1_{GCS}$ offspring.

File name: Supplementary Data 2

Description: Marker genes used for cell type identification in this study.

File name: Supplementary Data 3

Description: Differentially expressed genes (DEGs) and differentially abundant genes (DAGs) between $F1_{CON}$ and $F1_{GCS}$ groups.

File name: Supplementary Data 4

Description: scDRS analysis results for disease-associated gene sets.

File name: Supplementary Data 5

Description: Primer sequences used in this study.
