# Supplementary material for: Gestational choline supplementation regulates hippocampal granule neuron development and emotion-like behavior
Source: Commun Biol. 2026 Apr 2;9:731. doi: 10.1038/s42003-026-09955-7 (PMC13219442; doi:10.1038/s42003-026-09955-7)
Supplement: Supplementary file 8 — Supplementary data 5 [file 42003_2026_9955_MOESM8_ESM.docx]

**Primers used in this study**

| Experiments | Gene Symbols | Sequences |
| --- | --- | --- |
| For RT-qPCR | *Actin* | Forward primer 5′-ATGACCCAAGCCGAGAAGG-3′ |
|  |  | Reverse primer 5′-ATGACCCAAGCCGAGAAGG-3′ |
|  | *Gria1* | Forward primer 5′-GAGGTCCCGTAAACCTAGCG-3′ |
|  |  | Reverse primer 5′-GCTCAGAGCACTGGTCTTGT-3′ |
|  | *Gria2* | Forward primer 5′-GCCTTGCGACACCATGAAAG-3′ |
|  |  | Reverse primer 5′- ACACATCAGGGTAGGTGGGA-3′ |
|  | *Gria4* | Forward primer 5′-CAAAGGAGAATGTGGCAGCG-3′ |
|  |  | Reverse primer 5′-AAGCCGCCAACCAGAATGTA-3′ |
|  | *Grik1* | Forward primer 5′-TTGTTCTGGCTGCAGGACTC-3′ |
|  |  | Reverse primer 5′-GGAGTTGGTCGGATGGGTTT-3′ |
|  | *Grik2* | Forward primer 5′-GCTCTGAGAGGACTACCCCA-3′ |
|  |  | Reverse primer 5′-CTTCCACTGCAAGGGATGGT-3′ |
|  | *Grik5* | Forward primer 5′-CCTTGTCCTCCGTAAGGCTTC-3′ |
|  |  | Reverse primer 5′-AAAGCCCAGGATGTTGGAGG-3′ |
|  | *Grm1* | Forward primer 5′-GCCTTGCACCGTCTGATTTG-3′ |
|  |  | Reverse primer 5′-CCATAAGCTGGACGCTGAGT-3′ |
|  | *Grm2* | Forward primer 5′-ACCTTGGTCAAGGGTCTGGA-3′ |
|  |  | Reverse primer 5′-CCATGGAAGAGGGTCTACGC-3′ |
|  | *Grm5* | Forward primer 5′-CAGCTTAGATCGCAGCCACT-3′ |
|  |  | Reverse primer 5′-GGGTAAAATCACCAGGTGCG-3′ |
|  | *Grin2a* | Forward primer 5′-GGGACAGTACCCAATGGAAGT-3′ |
|  |  | Reverse primer 5′-GCGTCCAACTTCCCAGTTTT-3′ |
|  | *Grin2b* | Forward primer 5′-CCTCCTGTGTGAGAGGAAAGA-3′ |
|  |  | Reverse primer 5′-GTGGTCATTCCCAAAGCGTC-3′ |
|  | *Chrna3* | Forward primer 5′-CTCAGCTGGTGAAGGTGGATGA-3′ |
|  |  | Reverse primer 5′-TTCTCTGCAGGGACTCGCAT-3′ |
|  | *Creb3l2* | Forward primer 5′-CTCCTCATGCCAGACGCTTAT-3′ |
|  |  | Reverse primer 5′-GTTGATCCACGGAGGCTTCT-3′ |
|  | *Itpr3* | Forward primer 5′-ATCAGCACTTTGGGGCTGG-3′ |
|  |  | Reverse primer 5′-TCTGTGCAGAGTAGCGGTTC-3′ |
|  | *Pla2g4b* | Forward primer 5′-TGTGGACTTGGTCTCATGGC-3′ |
|  |  | Reverse primer 5′-GTCAGAGGAGGTTACTAGGTCC-3′ |
|  | *Slc1a1* | Forward primer 5′-CGCCGTGGTACTAGGAATTGT-3′ |
|  |  | Reverse primer 5′-ATGCTGGATACGATCAGCGG-3′ |
|  | *Slc1a2* | Forward primer 5′-TGCCCAAGCAGGTAGAAGTG-3′ |
|  |  | Reverse primer 5′-GCTCCCAGGATGACACCAAA-3′ |
|  | *Slc1a3* | Forward primer 5′-CAGTCTCGTCACAGGAATGGC-3′ |
|  |  | Reverse primer 5′-TTCCGGGGTGGATGATGATG-3′ |
|  | *Slc17a6* | Forward primer 5′-AACTTCTGCAGGAGCTGGAC-3′ |
|  |  | Reverse primer 5′-ACAACATGCCAACCTTGCTG-3′ |
|  | *Slc17a7* | Forward primer 5′-CTGGCAACGACAGCCTTTTG-3′ |
|  |  | Reverse primer 5′-GATCCCGAAGCTGCCATAGA-3′ |
|  | *Mif* | Forward primer 5′-GTGAACACCAATGTTCCCCG-3′ |
|  |  | Reverse primer 5′-GTGCACTGCGATGTACTGTG-3′ |
|  | *Ackr3* | Forward primer 5′-CTCAGCACTGAAGGAGCCTG-3′ |
|  |  | Reverse primer 5′-TTCCTGGGAACCACACCTCA-3′ |
|  | *Cd74* | Forward primer 5′-GGATGGCGTGAACTGGAAGA-3′ |
|  |  | Reverse primer 5′-CCTGGCACTTGGTCAGTACTTT-3′ |
|  | *Crcx2* | Forward primer 5′-TCGTAGAACTACTGCAGGATTAAG-3′ |
|  |  | Reverse primer 5′-GGGACAGCATCTGGCAGAATA-3′ |
|  | *Crcx4* | Forward primer 5′-GTGCCAGCCCCTAGATATACA-3′ |
|  |  | Reverse primer 5′-TTTTCATCCCGGAAGCAGGG-3′ |
|  | *Dcx* | Forward primer 5′-CAAGGCACACGGCTTTCTTT-3′ |
|  |  | Reverse primer 5′-GTGGAACCACAGCAACTTTTCC-3′ |
|  | *Calb1* | Forward primer 5′-ATTTCGACGCTGACGGAAGT-3′ |
|  |  | Reverse primer 5′-CCAATCCAGCCTTCTTTCGC-3′ |
|  | *Gda* | Forward primer 5′-GAGCGACAGCGGCAAAATAG-3′ |
|  |  | Reverse primer 5′-AGGCCTGGCATGAAGAACTC-3′ |
