# Supplementary material for: Gestational choline supplementation regulates hippocampal granule neuron development and emotion-like behavior
Source: Commun Biol. 2026 Apr 2;9:731. doi: 10.1038/s42003-026-09955-7 (PMC13219442; doi:10.1038/s42003-026-09955-7)
Supplement: Supplementary file 9 — reporting-summary [file 42003_2026_9955_MOESM9_ESM.pdf]

nature portfolio

Corresponding author(s): DBPR, COMMSBIO-25-7601C

Last updated by author(s): Mar 13, 2026

Reporting Summary

Nature Portfolio wishes to improve the reproducibility of the work that we publish. This form provides structure for consistency and transparency in reporting. For further information on Nature Portfolio policies, see our Editorial Policies and the Editorial Policy Checklist.

Statistics

For all statistical analyses, confirm that the following items are present in the figure legend, table legend, main text, or Methods section.

n/a	Confirmed	
☐	☒	The exact sample size (*n*) for each experimental group/condition, given as a discrete number and unit of measurement
☐	☒	A statement on whether measurements were taken from distinct samples or whether the same sample was measured repeatedly
☐	☒	The statistical test(s) used AND whether they are one- or two-sided<br>*Only common tests should be described solely by name; describe more complex techniques in the Methods section.*
☒	☐	A description of all covariates tested
☐	☒	A description of any assumptions or corrections, such as tests of normality and adjustment for multiple comparisons
☐	☒	A full description of the statistical parameters including central tendency (e.g. means) or other basic estimates (e.g. regression coefficient) AND variation (e.g. standard deviation) or associated estimates of uncertainty (e.g. confidence intervals)
☐	☒	For null hypothesis testing, the test statistic (e.g. *F*, *t*, *r*) with confidence intervals, effect sizes, degrees of freedom and *P* value noted<br>*Give P values as exact values whenever suitable.*
☒	☐	For Bayesian analysis, information on the choice of priors and Markov chain Monte Carlo settings
☒	☐	For hierarchical and complex designs, identification of the appropriate level for tests and full reporting of outcomes
☐	☒	Estimates of effect sizes (e.g. Cohen's *d*, Pearson's *r*), indicating how they were calculated

Our web collection on statistics for biologists contains articles on many of the points above.

Software and code

Policy information about availability of computer code

Data collection	No software was used to collect the data.
Data analysis	trim_galore v0.6.10, TopHat2 v2.1.1, StringTie v2.1.5, DESeq2 v1.30.1, PISA v0.2, STAR v2.7.4a, DoubletFinder v2.0.3, Seurat v4.4.0, R v4.0.2, R v4.3, CellChat v2.1.2, scDRS v1.0.3, chromap v0.2.4, d2c v1.4.4, ArchR v1.0.2, GraphPad Prism9.0

For manuscripts utilizing custom algorithms or software that are central to the research but not yet described in published literature, software must be made available to editors and reviewers. We strongly encourage code deposition in a community repository (e.g. GitHub). See the Nature Portfolio guidelines for submitting code & software for further information.

Data

Policy information about availability of data

All manuscripts must include a data availability statement. This statement should provide the following information, where applicable:

- Accession codes, unique identifiers, or web links for publicly available datasets
- A description of any restrictions on data availability
- For clinical datasets or third party data, please ensure that the statement adheres to our policy

All raw data have been deposited in the Genome Sequence Archive (https://ngdc.cncb.ac.cn/gsa/) at the BIG Data Center, Beijing Institute of Genomics, Chinese Academy of Sciences, under the accession number PRJCA043148 (CRA027938, shared link: https://ngdc.cncb.ac.cn/gsa/s/yV7I3H0p). The processed snRNA-Seq data

are available in OMIX, China National Center for Bioinformation / Beijing Institute of Genomics, Chinese Academy of Sciences (https://ngdc.cncb.ac.cn/omix; accession no. OMIX011041, shared link: https://ngdc.cncb.ac.cn/omix/preview/0vWjDBaH).

Research involving human participants, their data, or biological material

Policy information about studies with human participants or human data. See also policy information about sex, gender (identity/presentation), and sexual orientation and race, ethnicity and racism.

Reporting on sex and gender	NA
Reporting on race, ethnicity, or other socially relevant groupings	NA
Population characteristics	NA
Recruitment	NA
Ethics oversight	NA

Note that full information on the approval of the study protocol must also be provided in the manuscript.

Field-specific reporting

Please select the one below that is the best fit for your research. If you are not sure, read the appropriate sections before making your selection.

☒ Life sciences ☐ Behavioural & social sciences ☐ Ecological, evolutionary & environmental sciences

For a reference copy of the document with all sections, see nature.com/documents/nr-reporting-summary-flat.pdf

Life sciences study design

All studies must disclose on these points even when the disclosure is negative.

Sample size	To investigate the effect of GCS on the body weight of mouse offspring, body weight was measured and compared in 6 mice from the F1CON group and 7 mice from the F1GCS group. For behavioral tests—including locomotor activity monitoring (LM), open field test (OFT), elevated plus maze (EPM), light/dark box test (L/D box), and forced swim test (FST)—14 mice per group (F1CON and F1GCS) were used for each assay. For RNA sequencing (RNA-Seq), 3 mice per group were analyzed at each of three developmental time points: P0, P10, and P30. For single-nucleus RNA sequencing (snRNA-Seq) and single-nucleus ATAC sequencing (snATAC-Seq) at P60, the left hippocampus from each of six mice per group was used for snRNA-Seq library preparation, while the contralateral right hippocampus from the same animals was processed for snATAC-Seq, allowing paired within-subject comparisons across epigenomic and transcriptomic layers. During quality control, one sample was excluded from both sequencing datasets due to insufficient library yield or poor nuclear integrity, resulting in five high-quality biological replicates per group for both assays. For qRT-PCR analysis, 3 mice per group were examined at P0 and P60. For western blotting, sample sizes were n = 6 per group at P0 and n = 3 per group at P60. In total, 43 male mice from each group (F1GCS and F1CON) were used across all experiments.
Data exclusions	For RNA-Seq data, sequencing adapters and low-quality reads were removed using the Trim Galore program. For snRNA-Seq data, raw sequencing reads were filtered and demultiplexed using PISA (version 0.2); the nuclear count matrix was further filtered based on the following criteria: number of unique molecular identifiers (UMIs) > 500, number of detected genes between 250 and 12,000, and mitochondrial content < 20%. For snATAC-Seq data, nuclei with a transcription start site (TSS) enrichment score < 4 or fragment counts < 1,000 were filtered out; doublets were additionally removed using the filterDoublets function with the parameter filterRatio = 1.5.
Replication	For behavioral tests, each group consisted of 14 biological replicates. For bulk transcriptome sequencing, each group included 3 biological replicates. For single-nucleus omics analyses, each group comprised 5 biological replicates. For qRT-PCR, each group was represented by 3 biological replicates across three developmental stages. For western blotting, each group contained 3–6 replicates at P0 and P60.
Randomization	To avoid litter effects, only 1–2 male offspring per dam were used for each of the omics sequencing analyses and behavioral experiments.
Blinding	Since all our analyses were performed with known samples, this study does not apply to blinding.

Reporting for specific materials, systems and methods

We require information from authors about some types of materials, experimental systems and methods used in many studies. Here, indicate whether each material, system or method listed is relevant to your study. If you are not sure if a list item applies to your research, read the appropriate section before selecting a response.

Materials & experimental systems

n/a	Involved in the study	
☐	☒	Antibodies
☒	☐	Eukaryotic cell lines
☒	☐	Palaeontology and archaeology
☐	☒	Animals and other organisms
☒	☐	Clinical data
☒	☐	Dual use research of concern
☒	☐	Plants

Methods

n/a	Involved in the study	
☒	☐	ChIP-seq
☒	☐	Flow cytometry
☒	☐	MRI-based neuroimaging

Antibodies

Antibodies used

For the target protein Doublecortin (DCX), the antibody was obtained from Immunoway (USA) with catalog number YM8640 and clone number PT0871R, used at a dilution of 1:6000; the lot number was not specified. The loading control antibody against β-Tubulin was sourced from Abclonal (China) with catalog number A12289 and used at a dilution of 1:10,000; neither the clone name nor lot number was provided. The secondary antibody for Western blotting was an HRP-linked anti-rabbit IgG from Cell Signaling Technology (USA) with catalog number 7074S, lot number 33, and used at a dilution of 1:5000; the clone name was not specified.

Validation

The primary antibody targeting Doublecortin (DCX) is a rabbit monoclonal antibody from Immunoway (Cat# YM8640, Clone PT0871R), which was used for Western blotting analysis of mouse hippocampal tissue. According to the product datasheet provided by Immunoway, this antibody has been validated for Western Blot (WB) and Immunohistochemistry (IHC) applications, with specific validation data for WB experiments on mouse tissue. The specificity of the antibody in the present study is supported by the Western blot data shown in Figures 3D and 3E, where a clear, single band is observed at the expected molecular weight of DCX in mouse hippocampal lysates.
The loading control antibody is a rabbit monoclonal anti-β-Tubulin from Abclonal (Cat# A12289), utilized as an internal reference for Western blotting of mouse hippocampal tissue. As indicated in Abclonal's product datasheet for A12289, this antibody is validated for WB, Immunofluorescence (IF), and IHC applications across multiple species including mice, with the manufacturer providing validation data confirming its reactivity with mouse samples. Its effectiveness is corroborated by the consistent and strong signals detected across all samples in Western blot experiments (Figures 3D and 3E), confirming its reliability as a loading control.
The secondary antibody is an HRP-linked anti-rabbit IgG from Cell Signaling Technology (Cat# 7074S, Lot# 33), used for detecting the aforementioned rabbit-derived primary antibodies in Western blotting. According to the product page for Cat# 7074S, this antibody has undergone extensive validation and been cited in over 18,553 publications . Specifically designed for Western blotting, it is purified and exhibits minimal cross-reactivity with immunoglobulins from other species under standard experimental conditions. The successful application of this secondary antibody is evidenced by the clear and specific detection of primary antibodies in the Western blot results presented in the manuscript.
Both primary antibodies are commercially available monoclonal antibodies with manufacturer-validated data for WB on mouse samples, and their specific utility in this study is further supported by the experimental data in the manuscript. The secondary antibody is a widely used, highly cited reagent that has been validated for Western blotting applications.

Animals and other research organisms

Policy information about studies involving animals; ARRIVE guidelines recommended for reporting animal research, and Sex and Gender in Research

Laboratory animals

Six-week-old male and female mice of the C57BL/6J strain were paired for mating and assigned to two groups: CON and GCS. Offspring derived from pregnant females in the F0f-GCS group were designated as the F1GCS group, while those from the F0f-CON group served as the F1CON group. All offspring were maintained on a standard diet after birth. Bulk RNA sequencing (RNA-Seq) was performed on offspring at postnatal days 0, 10, and 30 (P0, P10, P30). Mood-related behavioral tests, single-nucleus RNA sequencing (snRNA-Seq), and single-nucleus ATAC sequencing (snATAC-Seq) were conducted when offspring reached 2 months of age. Quantitative real-time PCR (qRT-PCR) and Western blot analyses were carried out at P0 and P60.

Wild animals

The study did not involve wild animals.

Reporting on sex

The results of this study on the offspring mice are applicable only to males.

Field-collected samples

The study did not involve samples collected from field.

Ethics oversight

All experiments were approved by the Animal Use Committee of the Institute of Zoology, Chinese Academy of Sciences, and in accordance with the guidelines of the National Institutes of Health (NIH).

Note that full information on the approval of the study protocol must also be provided in the manuscript.

Plants

Seed stocks	The study did not involve plants.
Novel plant genotypes	NA
Authentication	NA
